# Combined Effects of Heavy Metal and Simulated Herbivory on Leaf Trichome Density in Sunflowers

**DOI:** 10.3390/plants13192733

**Published:** 2024-09-29

**Authors:** Eyal Grossman, Ilana Shtein, Michal Gruntman

**Affiliations:** 1 Porter School of the Environment and Earth Sciences, Tel Aviv University, Tel Aviv 69978, Israel; eyalsun1@gmail.com; 2 Eastern R&D Center, Milken Campus, Ariel 40700, Israel; ilanash@ariel.ac.il; 3 School of Plant Sciences and Food Security, Tel Aviv University, Tel Aviv 69978, Israel

**Keywords:** *Helianthus annuus*, herbivore defense, jasmonic acid, leaf trichomes, metal hyperaccumulation, phytoremediation

## Abstract

Trichomes play a key role in both heavy metal tolerance and herbivory defense, and both stressors have been shown to induce increased trichome density. However, the combined effect of these stressors on trichome density in general, and specifically on metal-hyperaccumulating plants, has yet to be examined. The aim of this study was to test the effect of cadmium availability and herbivory on leaf trichome density and herbivore deterrence in the metal hyperaccumulator *Helianthus annuus*. To test this, *H. Annuus* plants were grown in control pots or pots inoculated with 10 mg/kg cadmium and were subjected to either no herbivory or simulated herbivory using mechanical damage and foliar jasmonic acid application. Herbivore deterrence was tested in a feeding assay using *Spodoptera littoralis* caterpillars. Interestingly, while the trichome density of *H. annuus* increased by 79% or 53.5% under high cadmium availability or simulated herbivory, respectively, it decreased by 26% when the stressors were combined. Furthermore, regardless of cadmium availability, simulated herbivory induced a 40% increase in deterrence of *S. littoralis*. These findings suggest that the combination of metal availability and herbivory might present excessive stress to hyperaccumulators. Moreover, they suggest that the risk of metal bioaccumulation in phytoremediation can be reduced by simulated herbivory.

## 1. Introduction

Heavy metals such as lead (Pb), arsenic (As), or cadmium (Cd) are not essential for the proper homeostasis of organisms, including animals and plants, and can be lethal in low concentrations if they accumulate in living tissues. However, some plant species, known as metal hyperaccumulators, can accumulate extremely high concentrations of heavy metals from the soil or water. These species can uptake and store metals in their shoots in concentrations that exceed those of the growth media without exhibiting signs of toxicity [1,2,3]. As a result, metal hyperaccumulators have been extensively studied for their potential application in the field of soil remediation, a method termed “phytoremediation” [1,4,5].

The selection pressures that may have driven plants to develop metal hyperaccumulation abilities have been studied over the years [2,6,7,8]. Currently, the hypothesis that has received the most empirical support is the “elemental defense hypothesis,” which posits that plants use heavy metals and metalloids as a defense strategy to deter herbivores and pathogens [2,6,8]. For example, several studies have shown that the accumulation of zinc (Zn), selenium (Se), and Cd in leaves can significantly reduce the feeding and survival of both specialist and generalist leaf herbivores [9,10,11,12,13]. Moreover, for some species, metal hyperaccumulation was found to be a constitutive trait expressed in all populations, such as the Zn hyperaccumulators *Noccaea caerulescens* and *Arabidopsis halleri*, but for others, such as the Cd hyperaccumulator *Sedum allferdi*, it was found to be a facultative trait expressed only in populations growing in soils with elevated metal concentrations (metallicolous populations) [2,8,14,15]. Interestingly, a few recent studies have demonstrated that the accumulation of heavy metals can also be induced by herbivore damage, as well as by simulated herbivory [16,17,18].

Metal hyperaccumulators can accumulate extreme concentrations of heavy metals due to their high metal tolerance capacity, which is attributed to the compartmentalization and sequestration of metals in special organs, such as vacuoles, found in leaf epidermis and mesophyll cells and trichomes [2,15,19]. For example, leaf trichomes in the hyperaccumulator *Alyssum murale* have been shown to simultaneously sequester a few heavy metals, such as manganese, nickel, and cobalt [20,21]. Furthermore, in *Arabidopsis halleri* and *Brassica juncea*, the highest concentrations of heavy metals in the leaves can be found in the trichomes rather than the other epidermal cells [22,23]. Interestingly, trichomes were also shown to play a role in the detoxification of heavy metals in non-hyperaccumulators such as *Nicotiana tabacum* and *Arabidopsis thaliana* [24,25,26]. Moreover, although yet relatively unexplored, a few studies have shown that leaf trichome density can also increase in response to elevated soil metal concentrations in a few species that can accumulate moderate metal concentrations [19,25,27,28,29]. For example, leaf trichome density in *N. tabacum* seedlings was shown to double under Cd stress [25]. These findings suggest that trichome density is a plastic trait that can be induced to facilitate the tolerance of elevated heavy metal concentrations.

In addition to facilitating metal tolerance, trichomes are known to play an important role in herbivore defense [30,31,32,33]. Trichomes can be broadly divided into glandular or non-glandular trichomes. Glandular trichomes provide plants with chemical defenses and can produce, store, and secrete secondary metabolites such as terpenes and flavonoids, designed to repel herbivores and hinder their movement, as well as to attract mutualist predators [31,33]. In contrast, non-glandular trichomes can act as a physical barrier against herbivores by wounding, preventing movement, and disrupting oviposition on the plant surface [31,32,34]. Moreover, the density of both glandular trichomes and non-glandular trichomes has been shown to increase following herbivore damage [30,33,35,36]. For example, herbivore damage was shown to increase trichome density in the leaves of *Salix cinerea* [35], while simulated herbivory using methyl jasmonate was shown to induce increased trichome density in the leaves of *Solanum lycopersicum* [36]. However, the effect of herbivory on trichome density has yet to be studied in metal hyperaccumulators or metal-tolerant plants in general.

Trichome density can, therefore, play a key role in both metal tolerance and herbivore defense, and both heavy metal availability and herbivore damage might synergistically increase trichome density in plants. Moreover, this synergistic effect can be particularly adaptive if, as mentioned above, heavy metal uptake in hyperaccumulators is further induced in response to herbivory [16,18]. However, to the best of our knowledge, the combined effect of these two stressors on trichome density in plants has yet to be examined. Recently, simulated herbivory using leaf piercing and the exogenous application of the phytohormone jasmonic acid (JA) was shown to significantly increase Cd uptake in the leaves of *Helianthus annuus* [16]. The simulation of herbivory by JA exogenic application is a known practice used to induce and study defense responses in plants [18,37,38,39]. JA is involved in a variety of developmental processes, such as root growth, germination, and fruit ripening, and is mostly known to act as a signal to induce herbivory and pathogenic defense responses [40,41,42]. The fact that simulated herbivory, using JA, can induce metal uptake by plants suggests that this method can be used to accelerate the duration of the phytoremediation process, which is a key limitation in this field [4,43]. However, another limitation of phytoremediation is the risk of the biomagnification of heavy metals in higher trophic levels through the ecosystem, which can occur via herbivores feeding on contaminated plants [44]. Hence, if the application of simulated herbivory can also increase trichome density, it might be a valuable tool to repel herbivores and to reduce the biomagnification of metals, alongside accelerating the phytoremediation process.

The aim of this study was to test the effect of Cd availability and simulated herbivory on leaf trichome density and herbivore deterrence in *Helianthus annuus*. *H. annuus* is known for its metal hyperaccumulation abilities and is a species commonly used in phytoremediation studies [45,46,47,48]. Previous studies have demonstrated the involvement of *H. annuus*’s leaf trichomes in heavy metal tolerance and accumulation. For example, ref. [24,49] found that non-glandular trichomes in *H. annuus* can accumulate and detoxify manganese as its soil concentrations increase; moreover, [50] demonstrated that *H. annuus* can also sequester foliar-applied Zn fertilizer through non-glandular trichomes. Furthermore, the exogenic application of methyl jasmonate, a derivative of JA, has previously been shown to induce increased trichome density in *H. annuus* leaves [51,52] and, as previously mentioned, simulated herbivory using foliar-applied JA has been shown to induce increased leaf Cd uptake [16].

We hypothesized that Cd availability and simulated herbivory would act synergistically and induce increased trichome density (of both glandular and non-glandular trichomes) in *H. annuus*. We also hypothesized that, due to the combined effect of simulated herbivory and Cd availability on trichome density, it will also result in the highest herbivore deterrence levels. While there is abundant research regarding the effect that heavy metals and herbivores have on trichome density, to date, there is no study that has tested the combined effect of these two stressors. By examining trichome density variation under both biotic and abiotic stress, we can gain a deeper understanding of plant defenses and decision making, as well as of ways to improve phytoremediation practices through herbivore deterrence and a reduced risk of biomagnification.

## 2. Results

### 2.1. Plant Performance

**Figure 1:**
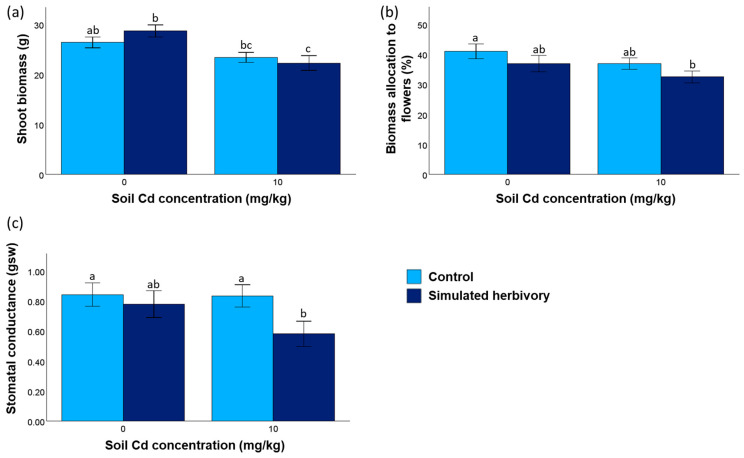
*H. annuus* performance (means ± SE), including (**a**) shoot biomass, (**b**) biomass allocation to flowers, and (**c**) stomatal conductance in response to soil Cd concentration and simulated herbivory treatments (N = 20 for each treatment combination). Different letters indicate statistically significant pairwise comparisons (LSD test, *p* < 0.05).

Soil Cd concentration had a negative effect on both shoot biomass and biomass allocation to flowers (Figure 1a,b; Table 1). For example, in the treatments without simulated herbivory, Cd availability caused an 11% decrease in shoot biomass and a 10% decrease in biomass allocation to flowers. Moreover, while simulated herbivory did not affect shoot biomass, it had a negative effect on both biomass allocation to flowers and stomatal conductance, particularly when the plants grew under high soil Cd concentrations (Figure 1b,c; Table 1). Under Cd availability, simulated herbivory caused biomass allocation to flowers to decrease by 12% and stomatal conductance to decrease by 30%.

### 2.2. Trichome Density

**Figure 2:**
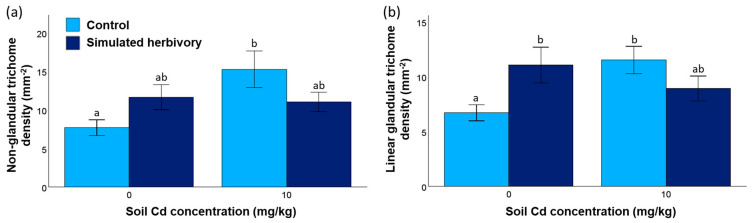
Densities (means ± SE) of (**a**) non-glandular trichomes and (**b**) linear glandular trichomes of *H. annuus* leaves in response to soil Cd concentration and simulated herbivory treatments (N = 7–8 leaf samples for each treatment combination). Different letters indicate statistically significant pairwise comparisons (LSD test, *p* < 0.05).

**Figure 3:**
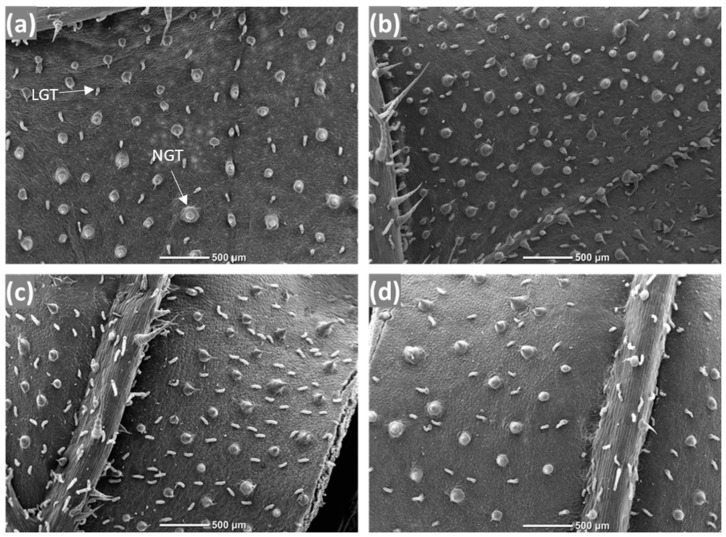
Examples for scanning electron microscope (SEM) imaging of *H. annuus* leaf surfaces, used to measure the densities of non-glandular trichomes (NGT) and linear glandular trichomes (LGT) from plants under treatment of (**a**) no herbivory and no Cd control; (**b**) simulated herbivory and no Cd; (**c**) no herbivory and Cd; and (**d**) simulated herbivory and Cd.

The density of non-glandular trichomes in the leaves of *H. annuus* increased in response to soil Cd concentration (Figure 2a and Figure 3; Table 2). However, Cd availability had no effect on the density of linear glandular trichomes (Figure 2b and Figure 3; Table 2). Overall, simulated herbivory had no effect on the density of either trichome type, but its effect depended on the soil Cd concentration (Figure 2 and Figure 3; Table 2: soil Cd concentrations × simulated herbivory interaction). Specifically, while trichome density increased in response to simulated herbivory when plants grew without the presence of Cd, it decreased in response to simulated herbivory when Cd was added to the soil (Figure 2 and Figure 3). For example, the density of linear glandular trichomes increased by over 60% due to simulated herbivory when *H. annuus* grew without Cd availability but decreased by over 20% with soil Cd concentrations (Figure 2b, Table 2).

### 2.3. Feeding Assay

**Figure 4:**
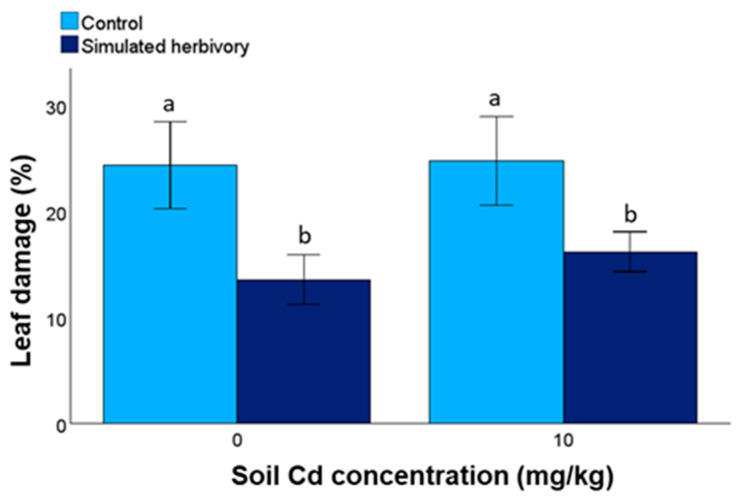
Leaf damage of *H. annuus* by *S. littoralis* in response to soil Cd concentration and simulated herbivory treatments (N = 17–20 feeding assays for each treatment combination). Different letters indicate statistically significant pairwise comparisons (LSD test, *p* < 0.05).

The leaf damage by *S. littoralis* caterpillars decreased in response to the simulated herbivory treatments but was not affected by soil Cd concentration (Figure 4; Table 3). In both the control and Cd treatments, the decrease in leaf damage due to the simulated herbivory treatment was about 40%.

## 3. Discussion

In this study we tested the combined effect of abiotic and biotic stresses on induced trichome density in *H. annuus* leaves. Specifically, we investigated whether linear glandular trichome and non-glandular trichome density changes in response to Cd soil concentration coupled with simulated herbivory in *H. annuus*. We predicted that trichome density, regardless of type, will increase under each stressor alone, as has been previously demonstrated [19,27,29,30,33,36], but would increase even more under both stressors combined due to the dual role of the trichomes in both metal detoxification and herbivore deterrence, as well as due to the positive effect of simulated herbivory on metal uptake in *H. annuus* [16]. Surprisingly, however, while each environmental stressor (simulated herbivory or Cd availability) induced increased trichome density as predicted, their combination had a negative effect on trichome density, regardless of their type.

*H. annuus* is known for its ability to accumulate high concentrations of Cd [45,47,48,53,54,55,56]. In a hydroponic setup, [56] showed that *H. Annuus* can accumulate over 50 mg/kg of Cd in its leaves. [45] demonstrated that *H. Annuus* shoots can accumulate Cd concentrations of up to 65.7 mg/kg, and [53] found that *H. Annuus* shoots were able to accumulate 448 mg/kg of Cd when plants grew in soil amended with multipool heavy metals. In a previous study with a similar setup, simulated herbivory was shown to induce a 39% increase in leaf Cd concentrations in *H. annuus* [16], suggesting it should have resulted in a similar increase in trichome density. In that study, simulated herbivory increased leaf Cd concentrations from 25.66 mg/kg to 35.76 mg/kg when the plants grew in soil with a Cd concentration of 10 mg/kg. However, in the current study, it is possible that, while the Cd leaf concentration increased due to simulated herbivory as a defense response, the use of JA might have also triggered other defense and stress responses that lead to resource allocation trade-offs between trichome production and those functions. The exogenic application of JA and its derivatives has previously been shown to induce various herbivory defense responses, including cell wall thickening, the production of secondary metabolites such as terpenes or glucosinolates, and extra floral nectar secretion [36,57,58,59,60,61,62,63,64,65,66]. On the other hand, under Cd availability, JA application may have also upregulated ion transport systems, activated antioxidant enzymes, and increased metal chelation capacity [63,64,65]. Generally, heavy metal accumulation involves several physiological processes, including the accumulation of metals from the soil into the roots, the translocation of metals from the roots to the shoots, and the sequestration of metals in the leaves [2]. These processes are energetically costly and might come at the expense of growth, reproduction, and other functions such as trichome production [65]. For example, while leaf trichome density of *Solanum viarum* was shown to increase in order to facilitate Pb, Cd, and Zn accumulation when the plants were faced with each metal alone, trichome density decreased when the plants grew in soil with multiple-metal stress [66]. It is evident from the performance variables measured in our study that the plants experienced the highest stress under the combination of Cd and simulated herbivory. For instance, while the shoot biomass was only affected by Cd, its allocation to flowers was most impaired under both Cd and simulated herbivory stress, suggesting that resources might have been diverted away from both growth and reproduction to tolerance and defense [67,68]. Moreover, stomatal conductance, which is often used as a stress (biotic or abiotic) indicator in plants [69], was only negatively affected under the dual stress. Usually, following herbivore damage, stomatal conductance decreases, since plants tend to close their stomata in order to reduce transpiration and conserve viable resources such as water, carbon, and nitrogen, which will be needed for repairing any damaged tissues and producing new ones [69,70]. Similarly, metal stress can often induce stomatal closure as part of the defense mechanism applied by plants to limit transpiration, which will reduce metal uptake [71,72,73]. However, in our study, stomatal conductance did not change under each stressor but only under both.

While trichomes are a major site for leaf heavy metal storage, other leaf tissues, such as epidermal cells, mesophyll cells, and even cuticle, have been shown to accumulate heavy metals [2,26,74], and both JA and its derivative methyl jasmonate have been shown to induce changes in leaf properties and nutrient balance [51,75]. Thus, it is possible that an increase in leaf Cd concentrations under the combined Cd and simulated herbivory stress resulted in a decrease in trichome density because of the allocation costs involved, and that the excess Cd was accumulated in other leaf cells rather than mainly in the trichomes. Also, it is important to note that, although trichome density under Cd and simulated herbivory stress was low when the stressors were combined, it remained higher than that of the control treatments, indicating a level of Cd uptake induced by JA. However, since Cd concentrations were not analyzed in this study, we cannot be certain whether trichome density decreased due to the stress associated with elevated Cd concentrations or as a defense response induced by JA. Hence, future studies should test trichome density variations under multiple levels of soil Cd and the analysis of metal concentrations alongside the defense responses induced by exogenous JA.

We hypothesized that the combination of Cd availability and simulated herbivory treatments will provide *H. annuus* with the highest herbivory deterrence rate compared to each stressor alone. Surprisingly, and in contrast with our predictions, only simulated herbivory, and not Cd availability or trichome density, induced increased deterrence of the generalist herbivore *S. littoralis* caterpillar. Assuming that *H. annuus* accumulated Cd in their leaves when grown with Cd availability, the result of this study contradicts the elemental defense hypothesis, which posits that plants use heavy metals as a defense strategy against herbivores [2,6,8]. While the elemental defense hypothesis has gained wide support, the effectiveness of metal accumulation against herbivores is strongly dependent on the type of plant, metal, and herbivore in question [10,76]. For example, [77] found that the accumulation of Cd by the Cd hyperaccumulator *S. alfredii* does not immediately deter aphids from attacking the plants; however, over time, the presence of Cd in the phloem sap causes toxicity and reduces the population of aphids attacking the plant. On the other hand, ref. [78] demonstrated how the generalist herbivore *Plutella xylostella* (diamondback moth) can evolve to tolerate and even thrive on Se-accumulated leaves of *Stanleya pinnata*. Similarly, being a generalist herbivore, it is possible that *S. littoralis* (used in our study) also evolved tolerance to Cd. [79] studied the effect of multigenerational exposure of *S. littoralis* to Pb and Cd and found that, with every generation, the caterpillars evolved to be more tolerant to the metals and more efficient in detoxification and excretion. Since the caterpillars used in our study were not obtained from the wild, but rather from a research institute, where they have been reared for multipool generations on *Ricinus communis* leaves, it is possible that, over time, tolerance to various stresses was achieved in this genotype.

Although the non-choice assay shows that the *S. littoralis* caterpillars were not deterred when *H. annuus* grew in Cd-contaminated soil, they were deterred from feeding when the plants were subjected to simulated herbivory alone and when it was coupled with Cd availability. Without the presence of Cd in the soil, simulated herbivory likely induced the production and sequestration of secondary metabolites common to *H. annuus*, such sesquiterpene lactones, designed to repel herbivores [36,80,81]. However, with the presence of Cd, it is possible that the deterrence was achieved by a joint effect of both Cd and secondary metabolites [2]. Indeed, it has been hypothesized that metal hyperaccumulators can use heavy metals in concert with secondary metabolites to reduce their cost of production while maintaining overall herbivory defense [2,10,82,83]. Also, the reduction in leaf linear glandular trichomes of *H. annuus* under this treatment can point to a joint effect between leaf Cd and secondary metabolites. Hence, the decrease in leaf damage when *H. annuus* grew in Cd-contaminated soil and was subjected to simulated herbivory could be the result of the synergic effect of both Cd and secondary metabolites.

While the results of this study show that trichome density increases under Cd availability, they show that it does not translate into herbivory deterrence in *H. annuus*. However, they do point to the effectiveness of deterrence when incorporating simulated herbivory treatment using JA. Although further research is needed to unravel the tradeoffs involved between various plants defenses, specifically metal uptake, trichome density, and secondary metabolites, these findings show that the risk of metal bioaccumulation in phytoremediation projects can be reduced by JA application, along with increasing metal uptake and reducing the time needed for soil remediation [16].

## 4. Materials and Methods

### 4.1. Soil Preparation

The soil for the experiment was a mixture of potting soil and sand at a 1:1 ratio, supplemented with slow-release fertilizer (Osmocote 14-14-14, ICL) at a ratio of 3 g:1 L. This soil mix was used to fill 80 2 L pots (1.5 kg dry weight per pot), which were placed on greenhouse benches, each on a 1.45 L plastic plate to collect excess water during the experiment. The pots were irrigated with drippers for 80 min until 75% of the water holding capacity (WHC) was reached. The pots were then spiked with control solutions (without Cd) and Cd solutions (10 mg/kg). The Cd solution was prepared by mixing 15 mg of CdCl_2_ (Sigma-Aldrich, St. Louis, MO, USA) in 0.5 mL of distilled water, which was added to 200 mL of tap water. For the control solution, only 0.5 mL of distilled water mixed with 200 mL of tap water was used. Both the control and Cd solutions were added to the pots (40 pots per solution) in order to reach 100% WHC. The pots were irrigated daily for seven days to homogenize the solutions before transplanting of *H. annuus* seeds.

### 4.2. The Experiment

A greenhouse experiment with natural light conditions and a mean temperature of 24 °C was conducted between July and October 2021 at Tel Aviv University. *H. annuus* seeds (Emek 6 cultivar) were acquired from the “Seeds Shaar Haamakim” company and germinated in trays for 14 days. Then, 80 seedlings were transplanted to the pots described above (1 plant per pot). Upon transplanting, the pots (with their water collecting plates) were placed in 20 blocks on the greenhouse benches with four plants per block, according to plant size, which was determined by height and leaf number. Each block contained all four treatment combinations of either simulated herbivory (control vs. simulated herbivory) or soil Cd concentration (0 vs. 10 mg/kg). The plants were left to grow in the pots for one week before the start of the simulated herbivory treatments. In total, the experiment consisted of 80 plants (2 soil Cd concentrations (0 vs. 10 mg/kg) × 2 simulated herbivory treatments (control vs. simulated herbivory) × 20 blocks).

Simulated herbivory was performed by mechanical leaf damage and was followed immediately by JA application to induce a strong defense response [16,39]. Mechanical leaf damage was performed with a toothpick by piercing eight holes per leaf in 25% of the leaves per plant. JA application was performed by spraying the whole plant with approximately 2 mL of a 1 mM JA solution. The JA solution was prepared by mixing 250 mg of JA (Sigma-Aldrich) with 1 mL ethanol, 250 mL demineralized water, and 2.5 mL Triton X-100 (0.1%) [16,39]. For the control treatments, the leaves were not pierced, and the entire plant was sprayed with a control solution of 1 mL ethanol, 250 mL demineralized water, 2.5 mL Triton X-100 (0.1%), and 2.5 mL HCl to obtain the same pH as that of the JA solution [16,39]. Regardless of the type of treatment, the plants were sprayed until all leaves and shoots were covered with the solutions.

The control and simulated herbivory treatments were applied in parallel, once a week, for a period of four weeks. Before the first simulated herbivory treatments, transparent filters (30 cm diameter, 100 cm height) were placed around each pot to mitigate the chances of cross contamination by JA and the spread of volatile organic compounds that might be produced by the plants. Once transplanted, the plants were irrigated daily with drippers to maintain 60% WHC and were grown for 11 weeks until harvest, when they were 91 days old.

### 4.3. Measured Variables

#### 4.3.1. Plant Performance

Upon harvesting, the shoots and flowers were separately placed in paper bags, and their biomass was weighed after oven drying for 48 h at 80 °C. Biomass allocation of the flowers was estimated as the percentage of flower biomass relative to the total shoot biomass.

Before the harvest, stomatal conductance was measured with a portable porometer system (LI-600, LI-COR, Lincoln, NE, USA) between the second and third simulated herbivory treatments when the plants were 30 days old. One measurement was taken per plant, from an undamaged leaf on the fifth or sixth node from the base, at midday (12:30–14:00).

#### 4.3.2. Trichome Density

The leaf samples were collected between the third and fourth simulated herbivory treatments when the plants were 40 days old. The leaf samples were collected prior to the flowering stage since metal-hyperaccumulating species and *H. annuus* have previously exhibited enhanced metal accumulation at the vegetative stage [54,56,58,84]. Only one mature, fully expanded, undamaged leaf was collected from the fifth or sixth node from the base of the shoot. In total, seven leaf samples were collected for each of the contaminated soil treatments, and eight samples were collected for each of the non-contaminated soil treatments. Mid-leaf fragments were immediately fixed in an FAA (70% ethanol: formaldehyde: glacial acetic acid 18:1:1) solution. For the SEM analyses, leaf fragments were dried in a Critical Point Dryer (Quorum K850, Quorum Technologies Ltd., Delaware, UK), glued on stubs, gold coated (Quorum SC7620, Mini S, Quorum Technologies Ltd., Lewes, UK [85]), and imaged on a scanning electron microscope (Hitachi TM3000 TableTop SEM, Hitachi High-Tech Corporation, Tokyo, Japan). The densities of both glandular and non-glandular trichomes were measured using ImageJ^®^ software, version number 1.54i 03 March 2024 [86] by counting their number within an area of 1 mm^2^ on the abaxial side of the leaf fragments (Figure 3). The glandular trichomes measured in this study are typically referred to as linear glandular trichomes [87,88,89], hence, from here on, we will use this term to describe glandular trichomes of *H. annuus*.

### 4.4. Feeding Assay

To study the effect of both simulated herbivory and Cd stress on herbivore deterrence of *H. annuus*, a feeding assay was performed using caterpillars of the generalist herbivore *Spodoptera littoralis* [89]. The *S. littoralis* caterpillars were supplied by the Plant Pathology and Weed Research unit at the Agriculture Research Organization, Volcani Center, where they were reared continuously on *Ricinus communis* leaves. Seven-day-old *S. littoralis* caterpillars were used in this study. Prior to the feeding assay, the caterpillars were fed *Lactuca sativa* leaves for 24 h and were kept at room temperature with a 12:12 h, light:dark cycle. The caterpillars were fed prior to the assay so as not to induce indiscriminate feeding [13,90]. The assay was conducted four weeks after the last simulated herbivory treatments, when plants were 70 days old. One leaf (between the fourth and sixth node from the base of the shoot) was removed from each plant and cut into a 20 cm^2^, which was placed in a Petri dish on moistened filter paper. An eight-day-old caterpillar was then placed on each square, and the Petri dishes were placed in the greenhouse with natural light conditions and a mean temperature of 24 °C. After 72 h, the leaf squares were photographed (Samsung, Galaxy A52 smart phone) and analyzed for leaf damage percentage by ImageJ software.

### 4.5. Data Analysis

The effects of soil Cd concentrations, simulated herbivory, and their interaction on the different dependent variables were analyzed using generalized linear mixed models (GLMM) with block as a random factor, except for the non-glandular trichomes and linear glandular trichomes, which were analyzed without block as a random factor, since the samples were collected based on the treatment type and not based on the individual plant. All analyses were performed with a normal distribution and an identity link function, except for biomass allocation to leaves and leaf damage percentage, which were measured with a normal distribution and a log link function. For analyses with significant effects, the differences between the treatment groups were analyzed using a post hoc pairwise LSD test. IBM SPSS Statistics 27 was used for all the statistical analyses.

## 5. Conclusions

Here, we examined for the first time the combined effect of both simulated herbivory using JA and heavy metals on trichome density in a species commonly used in phytoremediation systems. While each stressor induced enhanced trichome density, their combined effect was negative, resulting in a decrease of 26% and 25% for non-glandular and linear glandular trichomes, respectively. This decrease might have been the result of excessive stress; however, further studies are required to test this theory. Moreover, regardless of metal availability, simulated herbivory induced increased deterrence of a generalist herbivore, resulting in a 40% decrease in leaf damage. Although more studies are required in order to understand the trade-offs between different herbivory defense mechanisms, this study shows that simulated herbivory can be used in phytoremediation systems as a method that not only enhances metal uptake but also deters herbivores and reduces the risk of biomagnification

## Figures and Tables

**Table 1:** Results of generalized linear mixed models for the effects of soil Cd concentration (0 vs. 10 mg/kg), simulated herbivory (control vs. simulated herbivory), and their interaction on *H. annuus* performance, shoot biomass, biomass allocation to flowers, and stomatal conductance. Block was used as a random factor. Significant values are indicated in bold.

Fixed Effect			Shoot Biomass (g)		Biomass Allocation to Flowers (%)		Stomatal Conductance (gsw)	
	df_1_	df_2_	F	*p*	F	*p*	F	*p*
Soil Cd concentration (Cd)	1	76	18.827	**<0.001**	4.452	**0.038**	2.118	0.150
Simulated herbivory (SH)	1	76	0.29	0.592	4.487	**0.037**	5.009	**0.028**
Cd × SH	1	76	2.505	0.118	0.005	0.943	1.781	0.186
**Random effects**			**Wald Z**	***p***	**Wald Z**	***p***	**Wald Z**	***p***
Block			1.434	0.152	1.62	0.105	1.679	0.093

**Table 2:** Results of generalized linear mixed models for the effects of soil Cd concentration (0 vs. 10 mg/kg), simulated herbivory (control vs. simulated herbivory), and their interaction on the density (mm^−2^) of non-glandular trichomes and linear glandular trichomes. Significant values are indicated in bold.

Fixed Effect			Non-Glandular Trichome Density (mm^−2^)		Linear Glandular Trichome Density (mm^−2^)	
	df_1_	df_2_	F	*p*	F	*p*
Soil Cd concentration (Cd)	1	26	4.598	**0.042**	1.176	0.288
Simulated herbivory (SH)	1	26	0.006	0.938	0.499	0.486
Cd × SH	1	26	6.397	**0.018**	7.834	**0.01**

**Table 3:** Results of generalized linear mixed models for the effects of soil Cd concentration (0 vs. 10 mg/kg), simulated herbivory (control vs. simulated herbivory), and their interaction on *H. annuus*’s ability to deter leaf damage (%) caused by *S. littoralis*. Block was used as a random factor. Significant values are indicated in bold.

Fixed Effect			Leaf Damage (%)	
	df_1_	df_2_	F	*p*
Soil Cd concentration (Cd)	1	69	1.240	0.269
Simulated herbivory (SH)	1	69	16.706	**<0.001**
Cd × SH	1	69	0.44	0.509
**Random effects**			**Wald Z**	***p***
Block			2.368	**0.018**

## Data Availability

The data supporting the findings presented in this study are included in the Supplementary Materials.

## Supplementary Materials

The following supporting information can be downloaded at: https://www.mdpi.com/article/10.3390/plants13192733/s1. Table S1: Study’s dataset.
